# Positive Associations between Adipocyte Fatty Acid-Binding Protein Level and Central Arterial Stiffness in Peritoneal Dialysis Patients

**DOI:** 10.1155/2021/8849115

**Published:** 2021-02-06

**Authors:** Cheng-Hao Sung, Bang-Gee Hsu, Jen-Pi Tasi, Chih-Hsien Wang, Chiu-Huang Kuo

**Affiliations:** ^1^School of Medicine, Tzu-Chi University, Hualien, Taiwan; ^2^Division of Nephrology, Hualien Tzu Chi Hospital, Buddhist Tzu Chi Medical Foundation, Hualien, Taiwan; ^3^Division of Nephrology, Department of Internal Medicine, Dalin Tzu Chi Hospital, Buddhist Tzu Chi Medical Foundation, Chiayi, Taiwan; ^4^School of Post-baccalaureate Chinese Medicine, Tzu Chi University, Hualien, Taiwan

## Abstract

**Background:**

Adipocyte fatty acid-binding protein (A-FABP) plays essential roles in lipolysis, insulin resistance, and atherosclerosis. This study aimed to evaluate the relationship between serum A-FABP levels and carotid-femoral pulse wave velocity (cfPWV) in peritoneal dialysis (PD) patients.

**Methods:**

This study obtained fasting blood samples from 76 PD patients. A validated tonometry system was used to measure cfPWV. Patients with cfPWV values >10 m/s were classified into the high arterial stiffness group, whereas patients with values ≤10 m/s were classified into the low arterial stiffness group, according to the ESH-ESC 2013 guidelines. Serum A-FABP levels were measured using a commercial enzyme-linked immunosorbent assay kit.

**Results:**

Twenty-five (32.9%) of the 76 PD patients were classified in the high arterial stiffness group. Compared with the patients in the low arterial stiffness group, the high arterial stiffness group was older (*P* = 0.002) and had a longer PD vintage (*P* = 0.011), higher diastolic blood pressure (DBP, *P* = 0.036), higher fasting glucose levels (*P* = 0.012), higher serum C reactive protein levels (*P* = 0.001), and higher serum A-FABP levels (*P* < 0.001). A multivariate logistic regression analysis of the factors significantly associated with central arterial stiffness revealed that A-FABP (odds ratio (OR): 1.165, 95% confidence interval (CI): 1.056–1.284, *P* = 0.002), age (OR: 1.423, 95% CI: 1.153–1.757, *P* = 0.001), PD vintage (OR: 1.049, 95% CI: 1.015–1.085, *P* = 0.005), and DBP (OR: 1.152, 95% CI: 1.033–1.285, *P* = 0.011) were independent predictors of central arterial stiffness in PD patients. Furthermore, serum A-FABP levels (*β* = 0.476, adjusted *R*^2^ change: 0.197, *P* < 0.001) were significantly positively correlated with cfPWV according to the multivariable forward stepwise linear regression analysis.

**Conclusions:**

A-FABP levels are an independent marker of central arterial stiffness in PD patients.

## 1. Introduction

Arterial stiffness describes the rigidity of an artery and its inability to expand and contract during pressure changes; this condition is a common consequence of diabetes and chronic kidney disease [1, 2]. Accelerated arterial stiffness is more pronounced in patients with chronic hemodialysis than in the general population and plays a crucial role in cardiovascular mortality [1]. Unlike the hemodialysis group, peritoneal dialysis (PD) patients suffer from a greater accumulation of glycation end products (AGEs), which alters the collagen and elastin structure in the vessel wall and accelerates vascular calcification [2].

Adipocyte fatty acid-binding protein (A-FABP), also known as FABP-4, is an intracellular lipid chaperone molecule mainly secreted by adipocytes and macrophages [3]. A-FABP plays an essential role in obesity, lipolysis, insulin resistance, inflammation, atherosclerosis, and cardiac dysfunction [4]. When renal clearance decreases, A-FABP serum levels are 20 times higher in hemodialysis patients than in people with normal renal function [5]. Limited studies have evaluated A-FABP and vascular consequences in PD patients. Thus, the principal purpose of this study was to examine the correlation between arterial stiffness and A-FABP levels in PD patients.

## 2. Methods

### 2.1. Participants

From June 2015 to October 2016, 76 PD patients from Hualien and Dalin Tzu Chi Hospital who had undergone regular PD for more than three months were recruited. Among these subjects, 20 underwent four or five dialysate exchanges at night with an automated device (automated PD, APD) whereas the other 56 patients received continuous ambulatory PD (CAPD, Dianeal, Baxter Health Care, Taiwan), with three to five dialysate exchanges per day. Approval from the Research Ethics Committee, Hualien Tzu Chi Hospital, and Buddhist Tzu Chi Medical Foundation was obtained. All participants had provided their informed consent before participating in this study. Blood pressure (BP) was measured under standard mercury sphygmomanometers by trained staff in the morning with appropriate cuff sizes after resting for 10 minutes. Both systolic BP (SBP) and diastolic BP (DBP) were measured three times with an interval of 5 min between measurements. Hypertension was defined according to the Eighth Joint National Committee (JNC 8) guidelines (SBP ≥ 140 mmHg and/or DBP ≥ 90 mmHg or receiving any anti-hypertensive drugs in the past two weeks). Patients were defined as having diabetes mellitus (DM) if their fasting plasma glucose was more than 126 mg/dL or if they received oral hypoglycemic medications or insulin [6]. The exclusion criteria included previous malignancy, acute infection, acute myocardial infarction, pulmonary edema, and heart failure at the time of blood sampling. Data on the weekly total clearance of creatinine (Cre), peritoneal clearance of Cre, and fractional clearance index for urea (weekly Kt/V) were obtained from the patients' medical records.

### 2.2. Anthropometric Analysis

All anthropometric factors were measured in the morning, after overnight fasting, and without dialysate in the abdominal cavity. Height was measured to the nearest 0.5 cm, and body weight was measured to the nearest 0.5 kg in light clothing without shoes. Body mass index (BMI) was calculated as weight (kg) divided by height squared (m^2^) [7–11].

### 2.3. Biochemical Investigations

Biochemical parameters were determined before dialysis exchange in the morning after an 8-hour overnight fasting. Blood samples were immediately centrifuged at 3000 × g for 10 min. Serum samples were collected and stored at 4°C within one hour. Serum albumin, blood urea nitrogen (BUN), Cre, fasting glucose, triglyceride (TG), total cholesterol (TCH), total calcium, phosphorus, and C-reactive protein (CRP) levels were measured with an autoanalyzer (Siemens Advia 1800, Siemens Healthcare GmbH, Henkestr, Germany) [7–10]. Serum A-FABP (SPI-BIO, Montigny le Bretonneux, France) and intact parathyroid hormone (iPTH) levels (Diagnostic Systems Laboratories, TX, USA) were measured with a commercial enzyme immunoassay and enzyme-linked immunosorbent assay (ELISA) [8–11].

### 2.4. Carotid-Femoral Pulse Wave Velocity Measurements

**Figure 1:**
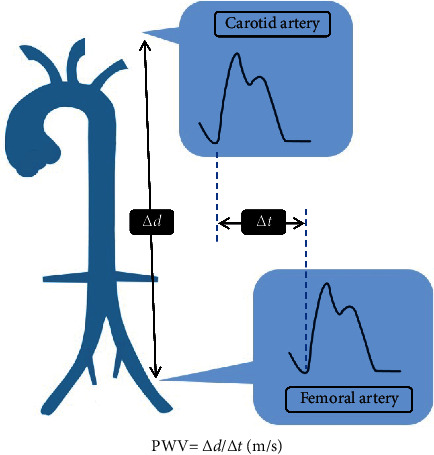
Carotid-femoral pulse wave velocity measurements. △d obtained by subtracting the distance from the carotid measurement site to the sternal notch and from the sternal notch to the femoral measurement site. △t obtained by time interval between the R waves from the ECG of carotid and femoral artery.

cfPWV was measured transcutaneously with applanation tonometry (SphygmoCor system, AtCor Medical, Australia) after recording blood pressure [8–11]. These measurements were performed with the patient in the supine position after 10 min of rest in a quiet and temperature-controlled room in the morning. Pulse wave recordings were performed consecutively at the carotid and femoral area. A simultaneous ECG signal provided the time interval between the *R* waves of carotid and femoral artery. Pulse wave recordings were performed consecutively at two superficial artery sites (carotid-femoral segment), with the distance obtained by subtracting the distance from the carotid measurement site to the sternal notch and from the sternal notch to the femoral measurement site. Using data from 10 consecutive cardiac cycles, the cfPWV was calculated based on the distance and mean time difference between the two recorded points (Figure 1). Quality indices, included in the software, were set to ensure data uniformity. According to the European Society of Hypertension (ESH) and the European Society of Cardiology (ESC), arterial stiffness was defined as cfPWV values >10 m/s in this study [12].

### 2.5. Statistical Analysis

The data were tested for normality using the Kolmogorov–Smirnov test. Data that were normally distributed were expressed as the mean ± standard deviation (SD), and comparisons between groups were performed using the Student's independent *t* test (two-tailed). Data that were non-normally distributed were expressed as medians and interquartile ranges, and comparisons between patients were performed using the MannWhitney *U* test (fasting glucose, TG, iPTH, and CRP). Data expressed as the number of patients were analyzed with the *χ*^2^ test. Variables that were significantly associated with arterial stiffness in PD patients were tested for independence in a multivariate logistic regression analysis (adapted factors: A-FABP, CRP, DBP, PD vintage, age, and fasting glucose). A-FABP levels correlated with cfPWV values were evaluated by simple linear regression in subgroup analysis with age, hypertension, DM, and gender. The data were analyzed using SPSS for Windows (version 19.0; SPSS Inc., Chicago, IL, USA). A *P* value <0.05 was considered statistically significant.

## 3. Results

The clinical manifestations of the 76 PD patients are listed in Table 1. Among these PD patients, 73.7% received CAPD. Comorbid conditions included hypertension (*n* = 65; 85.5%) and diabetes (*n* = 32; 42.1%). The medications reported by the patients included angiotensin-converting enzyme inhibitors (ACEi; *n* = 5; 6.6%), angiotensin-receptor blockers (ARB; *n* = 32; 42.1%), *β*-blockers (*n* = 28; 36.8%), calcium-channel blockers (CCB; *n* = 35; 46.1%), statins (*n* = 20; 26.3%), and fibrate (*n* = 3; 3.9%). There were no statistically significant differences in sex, PD model, ACEi, ARB, *β*-blockers, CCB, statins, or fibrate use between the two groups. Twenty-five PD patients (32.9%) were classified into the arterial stiffness group. The PD patients in the arterial stiffness group were older (*P* = 0.002) and had a longer PD vintage (*P* = 0.011), higher DBP (*P* = 0.036), higher fasting glucose levels (*P* = 0.012), higher CRP levels (*P* = 0.001), and higher serum A-FABP levels (*P* < 0.001) compared with the patients in the control group.

After adjusting for the factors significantly associated with arterial stiffness (age, PD vintage, fasting glucose, DBP, CRP, and A-FABP), the multivariate logistic regression analysis revealed that serum A-FABP levels (odds ratio (OR): 1.165, 95% confidence interval (CI): 1.056–1.284, *P* = 0.002), age (OR: 1.423, 95% CI: 1.153–1.757, *P* = 0.001), PD vintage (OR: 1.049, 95% CI: 1.015–1.085, *P* = 0.005), and DBP (OR: 1.152, 95% CI: 1.033–1.285, *P* = 0.011) were independent predictors of arterial stiffness in PD patients (Table 2).

**Figure 2:**
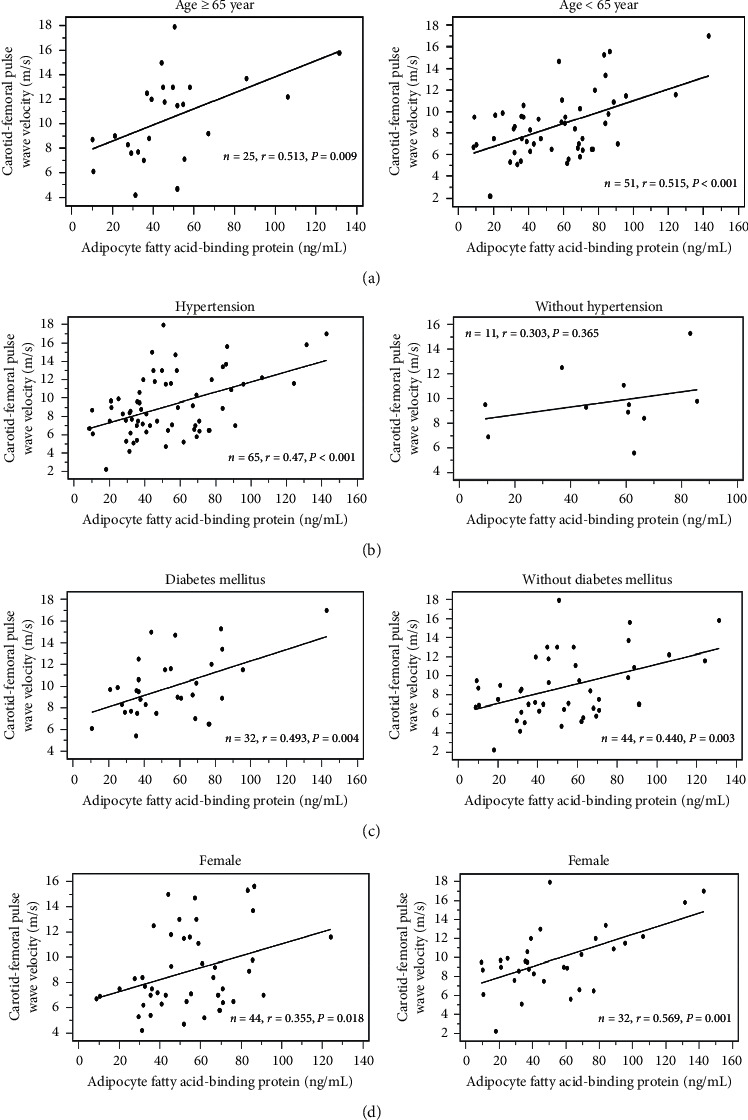
The scatter plot of A-FABP levels and cfPWV values in subgroup analysis with (a) older age, (b) hypertension, (c) DM, and (d) gender.

The cfPWV value was significantly positively correlated with age (*r* = 0.364; *P* < 0.001), PD vintage (*r* = 0.226; *P* = 0.049), logarithmically transformed glucose (log-glucose; *r* = 0.284; *P* = 0.013), log-CRP (*r* = 0.394; *P* < 0.001), and A-FABP levels (*r* = 0.456; *P* < 0.001) according to the simple linear regression analysis. After being analyzed by the multivariate stepwise linear regression analysis, age (*β* = 0.388, adjusted *R*^2^ change = 0.144, *P* < 0.001) and A-FABP levels (*β* = 0.476, adjusted *R*^2^ change = 0.197, *P* < 0.001) were significantly correlated with cfPWV values in PD patients (Table 3). Two-dimensional scattered plots of A-FABP levels and cfPWV values in subgroup analysis with older age, hypertension, DM, gender among these PD patients were drawn, which are presented as Figures 2(a)–2(d), respectively. Only PD patients without hypertension did not have significant differences in A-FABP levels and cfPWV values.

## 4. Discussion

The main finding of our study suggests that serum A-FABP levels are an independent predictor for arterial stiffness in PD patients after adjusting for other confounders. Age and A-FABP levels were significantly correlated with cfPWV values in PD patients.

Arterial stiffness in dialysis patients is thought to develop via two primary mechanisms: atherosclerosis in the intimal layer and arteriolosclerosis in the medial layer [13]. A-FABP was found in human atherosclerotic plaques and is involved in neointima formation after vascular injury in the endothelium [14, 15]. Several mechanisms have been proposed for the connection between A-FABP and atherosclerosis. A-FABP expressed in dendritic cells regulates the IKK-NF-*κ*B pathway and T-cell priming, which might contribute to atherosclerosis [16, 17]. In humans, A-FABP expression in macrophages increased in unstable carotid plaques of human endarterectomy samples [18]. Besides atherosclerosis, endothelial dysfunction, inflammation, and vascular smooth muscle differentiation play essential roles in arterial calcification. A-FABP could reduce the insulin-dependent nitric oxide synthase (NOS) expression in human umbilical vascular endothelial cells [19], while A-FABP inhibitor improves endothelial dysfunction in old ApoE-deficient mice [20]. Recombinant A-FABP increases gene expression of inflammatory markers in human coronary endothelial cells [14]. Moreover, A-FABP could induce coronary smooth muscle cell proliferation and migration [21].

According to previous studies, a high serum A-FABP level is associated with arterial stiffness measured by cfPWV in DM, hypertension with metabolic syndrome, and in geriatric subjects [8–10]. The serum A-FABP level is a predictor of cardiovascular events in patients with coronary artery disease and in patients with stable angina undergoing percutaneous coronary intervention [11, 22]. Our study showed that serum A-FABP levels were significantly associated with cfPWV values and were identified as independent predictors of arterial stiffness in PD patients. Beyond the insulin resistance, A-FABP could be a potential surveillance biomarker for arterial stiffness in the vulnerable population and provide early warning of poor cardiovascular outcomes.

Hypertension is a well-known independent and robust predictor of cardiovascular disease [23]. In addition to being present in coronary vessel beds, elevated blood pressure is also associated with significant calcification in the carotid, thoracic, abdominal, and iliac arteries as measured via electron-beam computed tomography [24]. High blood pressure increases vessel luminal pressure and stimulates collagen production, which destroys the balance of collagen and elastin [25]. However, numerous factors could alter the blood pressure among patients under regular hemodialysis, included dry bodyweight setting, overhydration status, and number of antihypertensive agents. In our result, the systolic and diastolic blood pressure was higher in the arterial stiffness group than in the control group, but there was no statistical difference between the two groups.

Age is an established clinical determinant of arterial stiffness. This correlation is independent of mean BP and other diseases in the general population such as diabetes [26]. Medial degeneration is an essential change during aging that results in the progressive stiffening of the large elastic arteries [27]. The accumulation of AGEs alters the protein properties, causes stiffness of the fibers and calcium deposition on the arterial wall, and leads to endothelial dysfunction triggered by an increase in oxidative stress given the correlation between age and vessel calcification [28, 29]. In our study, age and DBP were found to be possible risk factors for developing arterial stiffness in PD patients after adjusting for covariates.

The severity of arterial stiffness was proportional to the renal function decline and is predominant in dialysis patients [30, 31]. Chronic inflammation and calcifying inhibitor deficiency under a high calcium-phosphate burden status were precipitators for vascular calcification in end-stage renal disease, both in hemodialysis and PD [32, 33]. Limited literature has evaluated vascular changes over time in patients receiving PD. Chronic inflammation has been known as the precipitating factor for arterial stiffening. Recently, increasing literature reported the connection of A-FABP and inflammation effect [34, 35]. There is limited data about the interaction between CRP and AFABP. In our linear regression model, the regression coefficient of CRP multiplies A-FABP was very low, which means the interaction of CRP and A-FABP may not as important as the direct effect between PWV and AFABP.

The serum level of calcification inhibitors may differ in different dialysis modalities [36]. The residual renal function (RRF), which would gradually decline under renal replacement therapy, was independently correlated with the branchial-ankle pulse wave velocity value [37]. Furthermore, a small observational study of PD found that the heart-to-femoral pulse wave velocity increased even within one year [38]. In our study, PD vintage was another clinical factor associated with arterial stiffness according to the multivariate logistic regression analysis in PD patients.

Several limitations may have significantly influenced the results of our study. First, the cross-sectional nature of the study design and the limited number of participants in this study prevent us from being able to examine the causal relationship between serum A-FABP levels and arterial stiffness. Second, olmesartan use decreased serum A-FABP levels and ameliorated arterial stiffness as measured by the cardio-ankle vascular index in patients with hypertension [39]. Our study did not find a significant difference in arterial stiffness based on the antihypertensive drugs used, such as ACEi, ARB, *β*-blockers, and CCB in PD patients. Further long-term prospective studies are necessary to follow-up on the trend between serum A-FABP levels and arterial stiffness in PD patients.

## 5. Conclusion

In conclusion, we determined that serum A-FABP levels were higher in PD patients with arterial stiffness. Moreover, A-FABP may serve as an independent predictor for developing arterial stiffness and was significantly associated with cfPWV values in PD patients.

## Figures and Tables

**Table 1:** Clinical variable of the 76 peritoneal dialysis patients with or without arterial stiffness.

Characteristic	All participants (*n* = 76)	Control group (*n* = 51)	Arterial stiffness group (*n* = 25)	*P* value
Age (years)	55.51 ± 15.91	51.61 ± 16.12	63.48 ± 12.32	0.002^*∗*^
Peritoneal dialysis vintage (months)	50.83 ± 40.73	42.39 ± 37.74	68.04 ± 41.90	0.009^*∗*^
Height (cm)	160.04 ± 8.31	160.76 ± 8.24	158.56 ± 8.42	0.280
Body weight (kg)	63.18 ± 14.76	62.45 ± 13.16	64.66 ± 17.79	0.543
Body mass index (kg/m^2^)	24.70 ± 4.52	24.30 ± 4.16	25.51 ± 5.20	0.277
Carotid-femoral PWV (m/s)	9.26 ± 3.23	7.39 ± 1.67	13.08 ± 2.05	<0.001^*∗*^
Systolic blood pressure (mmHg)	143.03 ± 23.76	139.90 ± 24.75	149.40 ± 20.62	0.102
Diastolic blood pressure (mmHg)	84.50 ± 12.50	82.84 ± 12.99	87.88 ± 10.92	0.099
Total cholesterol (mg/dL)	169.08 ± 39.17	167.37 ± 37.67	172.56 ± 42.64	0.591
Triglyceride (mg/dL)	143.00 (93.75–222.25)	136.00 (91.00–209.00)	159.00 (108.00–231.00)	0.490
Fasting glucose (mg/dL)	106.00 (95.00–129.25)	102.00 (91.00–122.00)	117.00 (100.00–165.50)	0.012^*∗*^
Albumin (mg/dL)	3.72 ± 0.38	3.74 ± 0.42	3.69 ± 0.29	0.536
Blood urea nitrogen (mg/dL)	59.03 ± 18.43	59.31 ± 18.55	58.44 ± 18.54	0.848
Creatinine (mg/dL)	11.08 ± 3.14	11.12 ± 3.36	10.99 ± 2.71	0.876
Total calcium (mg/dL)	9.10 ± 0.77	9.07 ± 0.71	9.16 ± 0.90	0.637
Phosphorus (mg/dL)	5.23 ± 1.41	5.28 ± 1.46	5.12 ± 1.35	0.650
Intact parathyroid hormone (pg/mL)	247.16 (120.20–558.10)	266.55 (113.37–565.65)	229.20 (111.71–503.42)	0.822
C-reactive protein (mg/dL)	0.26 (0.07–0.91)	0.13 (0.06–0.53)	0.36 (0.26–1.36)	0.001^*∗*^
A-FABP (ng/mL)	53.55 ± 28.07	44.42 ± 22.01	72.17 ± 30.26	<0.001^*∗*^
Weekly (Kt/V)	2.09 ± 0.41	2.15 ± 0.44	1.97 ± 0.32	0.085
Peritoneal (Kt/V)	1.75 ± 0.47	1.75 ± 0.48	1.73 ± 0.46	0.845
Total clearance of creatinine (L/week)	59.26 ± 23.98	61.18 ± 26.10	55.34 ± 18.82	0.322
Peritoneal clearance of creatinine (L/week)	41.79 ± 16.10	41.34 ± 16.36	42.71 ± 15.83	0.729
Female, *n* (%)	44 (57.9)	31 (60.8)	13 (52.0)	0.466
Diabetes, *n* (%)	32 (42.1)	20 (39.2)	12 (48.0)	0.466
Hypertension, *n* (%)	65 (85.5)	43 (84.3)	22 (88.0)	0.668
CAPD, *n* (%)	56 (73.7)	36 (70.6)	20 (80.0)	0.381
ACE inhibitor use, *n* (%)	5 (6.6)	4 (7.8)	1 (4.0)	0.525
ARB use, *n* (%)	32 (42.1)	23 (45.1)	9 (36.0)	0.450
*β*-blocker use, *n* (%)	28 (36.8)	21 (41.2)	7 (28.0)	0.263
CCB use, *n* (%)	35 (46.1)	24 (47.1)	11 (44.0)	0.802
Statin use, *n* (%)	20 (26.3)	14 (27.5)	6 (24.0)	0.748
Fibrate use, *n* (%)	3 (3.9)	2 (8.0)	1 (2.0)	0.204

Values for continuous variables are shown as mean ± standard deviation after analysis by Student's *t* test; variables not normally distributed are shown as median and interquartile range after analysis by the Mann–Whitney *U* test; values are presented as number (%) and analysis after analysis by the chi-squared test. A-FABP, adipocyte fatty acid binding protein; CAPD, continuous ambulatory peritoneal dialysis; weekly Kt/V, weekly fractional clearance index for urea; ACE, angiotensin-converting enzyme; ARB, angiotensin-receptor blocker; CCB, calcium-channel blocker. ^*∗*^*P* < 0.05 was considered statistically significant.

**Table 2:** Multivariate logistic regression analysis of the factors correlated to aortic stiffness among 76 peritoneal dialysis patients.

Variables	Odds ratio	95% confidence interval	*P* value
Adipocyte fatty acid binding protein, 1 ng/mL	1.110	1.042–1.183	0.001^*∗*^
Age, 1 year	1.231	1.079–1.405	0.002^*∗*^
Peritoneal dialysis vintage, 1 month	1.029	1.007–1.051	0.010^*∗*^
Fasting glucose, 1 mg/dL	1.012	0.999–1.026	0.076
C Reactive protein, 1 mg/dL	1.580	0.586–4.261	0.366

Analysis of data was done using the multivariate logistic regression analysis (adopted factors: age, peritoneal dialysis vintage, fasting glucose, C reactive protein, and adipocyte fatty acid-binding protein). ^*∗*^*P* < 0.05 was considered statistically significant.

**Table 3:** Correlation between carotid-femoral pulse wave velocity levels and clinical variables among 76 peritoneal dialysis patients.

Variables	Carotid-femoral PWV (m/s)				
	Simple regression		Multivariate regression		
	*r*	*P* value	Beta	Adjusted *R*^2^	*P* value
Female	−0.140	0.228	–	–	–
Diabetes	0.173	0.135	–	–	–
Hypertension	−0.057	0.625	–	–	–
Age (years)	0.364	<0.001^*∗*^	0.388	0.144	<0.001^*∗*^
PD vintage (months)	0.226	0.049^*∗*^	–	–	–
Height (cm)	−0.007	0.949	–	–	–
Body weight (kg)	0.130	0.262	–	–	–
Body mass index (kg/m^2^)	0.149	0.198	–	–	–
Systolic blood pressure (mmHg)	0.123	0.288	–	–	–
Diastolic blood pressure (mmHg)	0.061	0.600	–	–	–
Total cholesterol (mg/dl)	0.019	0.870	–	–	–
Log-triglyceride (mg/dl)	0.082	0.481	–	–	–
Log-glucose (mg/dl)	0.284	0.013^*∗*^	–	–	–
Albumin (mg/dl)	−0.098	0.400	–	–	–
Blood urea nitrogen (mg/dl)	0.039	0.739	–	–	–
Creatinine (mg/dl)	−0.107	0.357	–	–	–
Total calcium (mg/dl)	0.006	0.961	–	–	–
Phosphorus (mg/dl)	−0.108	0.353	–	–	–
Log-iPTH(pg/ml)	−0.013	0.911	–	–	–
Log-CRP (mg/dl)	0.394	<0.001^*∗*^	–	–	–
A-FABP (ng/mL)	0.456	<0.001^*∗*^	0.476	0.197	<0.001
Weekly (Kt/V)	−0.153	0.188	–	–	–
Peritoneal (Kt/V)	−0.118	0.309	–	–	–
Total clearance of creatinine (l/week)	0.072	0.539	–	–	–
Peritoneal clearance of creatinine (l/week)	0.063	0.586	–	–	–

Data of triglyceride, glucose, CRP, and iPTH levels showed skewed distribution and therefore were log-transformed before analysis. Analysis of data was done using the simple regression analyses or multivariate stepwise linear regression analysis (adopted factors: PD vintage, age, log-glucose, log-CRP, and A-FABP). PD, peritoneal dialysis; A-FABP, adipocyte fatty acid binding protein; iPTH, Intact parathyroid hormone; CRP, C-reactive protein; weekly Kt/V, weekly fractional clearance index for urea. ^*∗*^*P* < 0.05 was considered statistically significant.

## Data Availability

The data underlying this study are available from the corresponding author on reasonable request.
